# Quantifying High-Performance Material Microstructure Using Nanomechanical Tools with Visual and Frequency Analysis

**DOI:** 10.1155/2018/4975317

**Published:** 2018-07-12

**Authors:** Emil Sandoz-Rosado, Michael R. Roenbeck, Kenneth E. Strawhecker

**Affiliations:** Composites and Hybrid Materials Branch, US Army Research Laboratory, Aberdeen, MD, USA

## Abstract

High-performance materials like ballistic fibers have remarkable mechanical properties owing to specific patterns of organization ranging from the molecular scale, to the micro scale and macro scale. Understanding these strategies for material organization is critical to improving the mechanical properties of these high-performance materials. In this work, atomic force microscopy (AFM) was used to detect changes in material composition at an extremely high resolution with transverse-stiffness scanning. New methods for direct quantification of material morphology were developed, and applied as an example to these AFM scans, although these methods can be applied to any spatially-resolved scans. These techniques were used to delineate between subtle morphological differences in commercial ultra-high-molecular-weight polyethylene (UHMWPE) fibers that have different processing conditions and mechanical properties as well as quantify morphology in commercial Kevlar®, a high-performance material with an entirely different organization strategy. Both frequency analysis and visual processing methods were used to systematically quantify the microstructure of the fiber samples in this study. These techniques are the first step in establishing structure-property relationships that can be used to inform synthesis and processing techniques to achieve desired morphologies, and thus superior mechanical performance.

## 1. Introduction

High-performance fibers have some of the highest specific stiffness and strength of any engineering material owing to the high orientation of polymer chains, making them uniquely suitable for demanding applications like body armor, cut-resistant fabric in space suits, deep-sea drill piping, aerospace composites, and so on [1]. Despite their incredible strength as bulk fibers, commercial high-performance fibers only achieve 15–30% of the intrinsic strength of their constituent molecules (in the case of ultra-high-molecular-weight polyethylene (UHMWPE) and Kevlar) after more than 40 years of research and development [2, 3]. The microstructure within a fiber, which is produced by the processing conditions during fiber production, governs the disparity between achieved strength and ideal molecular strength [4]. For further material advancements in high-performance fibers to occur, it is imperative to first establish structure-property relationships through careful quantification of microstructure, and later connect those relationships to processing conditions to target desirable fiber morphologies. To this end, this work establishes general analytical tools for the quantification of any high-performance material microstructure by any spatial scanning technique, as demonstrated by high-resolution atomic force microscopy (AFM) scans of ballistic fibers.

Widely used techniques for determining fiber morphology involve X-ray diffraction, infrared (IR) or Raman spectroscopy, and scanning or transmission electron microscopy (SEM or TEM) [4–13]. X-ray diffraction suffers from several limitations including averaging results over an entire interrogation volume (rather than being spatially resolved), assuming a material model a priori for analysis, and having wavelength-dependent resolution for detectable feature size. Similarly, IR and Raman spectroscopy can only report results averaged over a volume of a spot size that is limited by the wavelength and power of the interrogating laser (a minimum of cubic microns, generally) and analysis also requires assumptions about the material structure and its corresponding vibrational modes. Direct SEM is not particularly effective on nonconducting high-performance fibers like UHMWPE and Kevlar; a metal coating must be applied to the fibers to prevent melting by the electron beam, and the technique only reports topographical information. Finally, TEM, while able to provide spatial crystalline orientation, requires microtoming specimens to thicknesses of tens of nanometers which is difficult and potentially alters the microstructure due to violent cross sectioning [14].

Prior work has established high-resolution, transverse-stiffness AFM and careful shear notch sample preparation as a method for revealing internal fiber morphology that is rich with information [14–18]. This method is also known as the “FIB-notch” technique since a focused ion beam, FIB, is used to cut opposing notches along a fiber to induce shear failure along an internal plane via minimal applied load. Multifrequency atomic force microscopy (AFM) scans across these internal planes spatially resolve variations in material structure and transverse stiffness with subnanometer resolution, which is orders of magnitude higher than the other techniques. Furthermore, no material model is required a priori to extract data for AFM because the technique is performed in real space, rather than frequency space which is used for diffraction methods. Finally, AFM does not require thin samples, nor metal-coated samples, and can be performed on nearly any material, as demonstrated in this work by high-performance polymer fibers. The transverse stiffness measured by the AFM is particularly sensitive to changes in material phases, and can detect regions of amorphous polymer or crystalline polymer which are integral to fiber morphology. Given its relative simplicity and incredible resolution, AFM remains an ideal method for determining fiber microstructure. This work expands this powerful technique by adding rigorous and generalized methods of quantifying the observed morphologies for any material with a microstructure and any characterization technique that involves spatial mapping.

**Figure 1:**
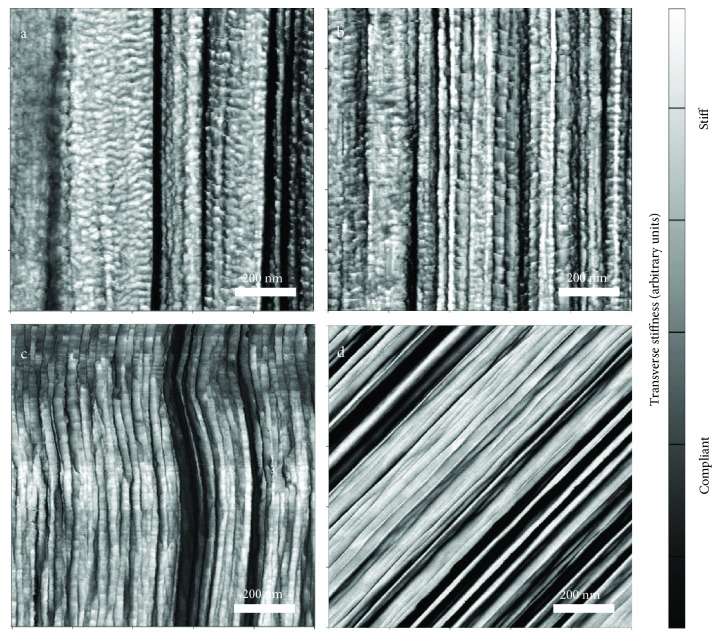
AFM transverse-stiffness scans of (a) S3000 commercial UHMWPE fiber, (b) SK76 commercial UHMWPE fiber, (c) K29 commercial Kevlar fiber, and (d) AuTx commercial para-aramid fiber. Dark shading corresponds to a relatively compliant structure, and light shading indicates a relatively stiff structure. The image in (b) was reprinted from [15] with permission from Elsevier.

The utility of unveiling the internal fiber structure using the FIB-notch sample preparation and AFM-stiffness mapping techniques was recently reported in a work comparing Kevlar fibers with varying processing histories [17]. In that work, strong direct relationships were made between the fiber microstructures and single-fiber tensile properties. The fiber microstructures were related back to the processing, and related to the fiber properties which govern performance. Through this technique the relationships between processing, structure, properties, and performance can be established, enabling a more effective and systematic design of higher performing ballistic fibers. Examples of the types of high-performance fibers that may be studied using this technique are shown in Figure 1 which includes 1 × 1-micron stiffness maps of the following materials: (a) Honeywell Spectra S3000, (b) DSM SK76, (c) DuPont™ Kevlar K49, and (d) Alchemie AuTx™.

## 2. Materials and Methods

### 2.1. High-Resolution Transverse Stiffness Atomic Force Microscope Scanning Methodology

In order to prepare the samples for these morphology studies, a novel FIB-notch technique was employed which has been detailed elsewhere [14]. Once the FIB-notch technique is used to expose an internal surface for scanning, the samples are mounted on a double-sided adhesive for AFM imaging.

**Figure 2:**
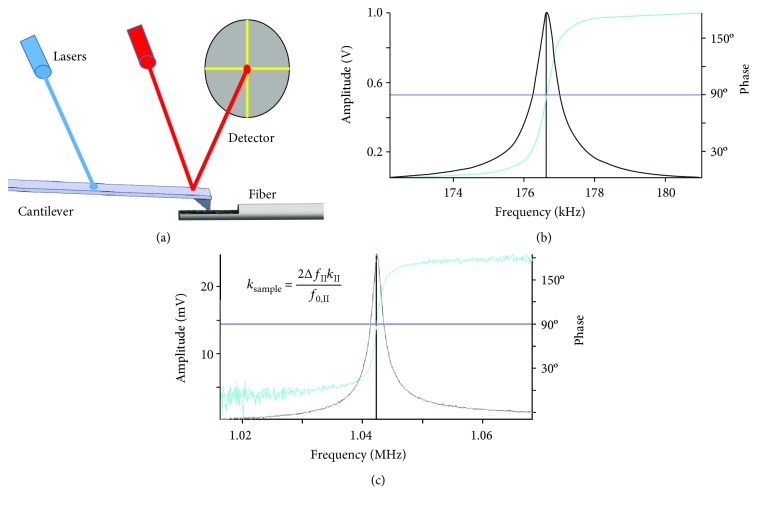
(a) AFM schematic with red sensing laser and blue vibrational excitation laser, (b) first resonant excitation for topographical AFM scanning, and (c) second, higher-frequency resonant excitation for transverse-stiffness measurements. Equation inlay shows the relationship between transverse stiffness of the sample, k_sample_, second-mode bending frequency of the AFM cantilever (shown as the peak in (c)), f_0,II_, second-mode bending stiffness of the AFM cantilever, k_II_, and measured change in second-mode frequency during scanning, Δf_II_.

In this work, specific techniques were added to standard imaging routines to enhance stiffness variations and decrease noise in the results. Namely, a laser is used to drive cantilever oscillation (blueDrive™) rather than a piezoelectric actuator, thereby decreasing noise. This is called photothermal excitation, where thermal heat causes a difference in expansion between the metal coating and cantilever, thereby leading to strain that bends the lever, and this method of excitation is superior to piezoacoustic excitation in that the data collected can be more accurately quantitatively interpreted [19].  Figure 2(a) is a schematic showing the setup which includes the typical cantilever, visible wavelength laser, and position-sensitive photodetector, as well as a blue wavelength laser for driving the cantilever at its first and second resonant frequencies. Because the coupling between the cantilever and the oscillation drive is direct and not mechanically initiated, the resulting resonant frequency curves (Figures 2(b) and 2(c), first and second normal bending eigenmodes, resp.) exhibit very low noise. Therefore, it can also be qualitatively seen how photothermal actuation contributes to good tracking and stiffness measurement with respect to the piezo driven analog.

Also, dual-frequency oscillation is used to isolate topography and stiffness variations [15, 16, 20]. Namely, AFM-stiffness imaging was utilized to produce a stiffness map from the tip probing the surface in the typical transverse (i.e., tapping) direction. To produce the image, the dual-frequency oscillation mode used is named (by Asylum Research) AM-FM mode [21] where the photothermal actuation of the lever occurs at two frequencies to excite the lever at its mode 1 and mode 2 bending frequencies simultaneously. The first bending mode (AM) is used in the feedback loop for standard AFM “tapping” or “AC mode” imaging. Here, the amplitude of oscillation is held constant by the *z*-feedback loop while the tip rises and falls to measure the topography (*z*-height) of the surface. The phase signal for the first bending mode feedback loop provides only qualitative nanomechanical information. The second bending mode is used to evaluate the tip-sample contact stiffness. This second mode operates at (typically) the mode 2 bending frequency, in frequency modulation (FM) mode using frequency feedback to hold the mode 2 phase at 90 degrees. Mode 2 operates at a much smaller amplitude than that of mode 1 and drive voltage for mode 2 is adjusted to keep the mode 2 amplitude constant. For mode 2, the relative frequency shift is assumed to be directly proportional to the shift in the tip-sample contact stiffness. Thermal tune techniques [22] are used to calculate the mode 1 spring constant. The mode 2 spring constant (*k*_II_ in Figure 2) before the tip contacts the sample, is calculated using the ratio of resonant frequencies and the stiffness of the first mode, according to the previously obtained relationship [20]. The relative mode 2 frequency shift (Δ*f*_II_/f_0,II_) provides an adjustment ratio, resulting in a quantitative tip-sample contact stiffness, as shown by the equation in Figure 2. Thus, the transverse stiffness at the sample surface can be mapped during scanning simultaneously with topography.

In practice, for this work, imaging was performed using a Cypher AFM with an ARC2 controller (Asylum Research). Commercial AFM tips (AC200TS, Asylum Research) were used as received. The nominal spring constants, k_I_ and k_II_ are 9 N/m and 200 N/m, respectively. The nominal tip radius is 7 nm. The typical oscillation amplitudes used here are on the order of 100 nm and 1 nm for mode 1 and mode 2, respectively [16].

## 3. Results and Discussion

### 3.1. Analytical Tools for Quantifying High-Performance Material Morphology

High-performance fibers derive their immense stiffness and strength from one of two principal material organization strategies: either they are composed of small molecules (100–200 nm long) [23] that have strong intermolecular hydrogen bonding, as in the case of Kevlar and other aramid fibers, or they consist of long molecules (1–10 *μ*m long) that have little intermolecular interaction, but orient and entangle slightly, as in the case of UHMWPE. In the case of the Kevlar aramid fibers, the small, rigid molecules are processed in a liquid crystal state and extruded to orient the molecules along the fiber axis where they coalesce into crystallites that are typically about 5 nm wide and tens of nm long [5, 6, 12]. These crystallites form a radially arranged pleated-sheet structure that repeats along the principal axis [11]. Conversely, the UHMWPE fiber is processed as a gel and extruded, heated, and elongated to achieve orientation. Depending on the processing conditions, UHMWPE can have lamellar crystallite structures of varying sizes that have repeat distances on the order of tens of nanometers [4, 13, 15]. For both aramid and polyethylene fibers, the constituent morphologies are repeated along and perpendicular to the main fiber axis. High-resolution AFM transverse-stiffness scanning yields images of high-performance fibers with excellent contrast between morphologies like highly crystalline regions and regions that are amorphous or have a large concentration of defects such as chain ends. These spatially-resolved AFM scans contrasting crystalline and disordered regions lend themselves to an array of visual analysis techniques that can be used to directly quantify the morphologies in a variety of ways such as crystallite density, geometry, and orientation.

Furthermore, the repeating nature of the morphologies of high-performance fibers make them ideal for quantification using frequency domain techniques such as fast Fourier transforms (FFT) or power density spectrum analyses, such as Welch's method. These analysis techniques can be applied on cross sections along or perpendicular to the fiber axis, and can be used to extract morphological repeat distances which correlate to crystallite and microfibril geometry. Unlike diffraction characterization techniques which report values averaged over the interrogation volume, frequency analysis applied to high-resolution AFM scans can be performed in spatial directions and locations, such as determining crystalline repeat lengths as a function of radial position. In the case of UHMWPE, quantifying the crystallite dimensions can give the percentage composition of crystalline and amorphous volume within the fiber and for Kevlar, determining crystallite repeat distances can potentially give information on molecular chain end density. These quantifications of polycrystalline morphology of high-performance fibers are critical for predicting mechanical properties at a larger scale.

#### 3.1.1. Visual and Frequency Analysis for Microstructure Identification

**Figure 3:**
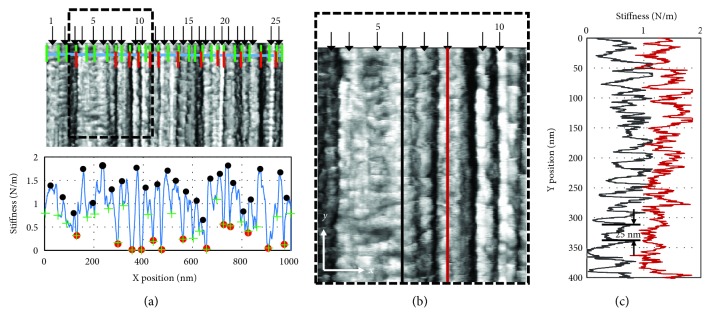
(a) Transverse-stiffness AFM scan of SK76 UHMWPE with black arrows identifying individual fibrils with the fibril number expressed every five fibrils, green “+” symbols indicating local minima for identifying the limits of a fibril, red “|” symbols indicating the minima used identifying fibril bundles, and the blue line indicating the profile which is expressed in the graph below which has the corresponding symbols (black dots correspond to the black arrows). The dashed black line indicates the area that is magnified in (b) which has vertical red and black lines with matching stiffness profiles in (c). AFM scans were reprinted from [15] with permission from Elsevier.

A typical metric for fibers is the fibril width measurement which can be illustrated using UHMWPE fibers such as the one in Figure 3(a) (a region of the SK76 fiber shown in Figure 1(b)). Transverse-stiffness AFM scans are uniquely suited for spatial measurements of fiber features because of the sharp contrast it provides for fiber morphologies. First, the fiber is scanned to capture the representative stiffness map, preferably with the fibrils arranged in a nearly vertical direction within the image. Stiffness as a function of position along the radial fiber direction is plotted as a line profile shown in Figure 3(a). The line profile can be compared with the image, and the darkest (most compliant) lines in the image will appear as low local minima in the line profile. In this case, it is beneficial to make the comparison between the image and the profile so that the perceived fibrillar interfaces in the image can be selected on the line profile.

**Figure 4:**
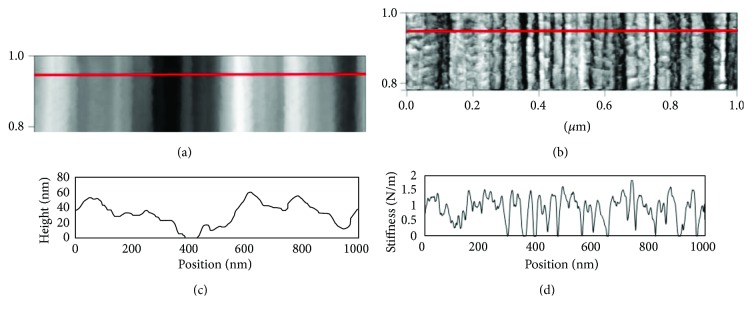
AFM (a) topography scan of SK76 UHMWPE fiber, (b) corresponding transverse-stiffness scan of the exact same area, (c) corresponding height profile along the red line in (a), and (d) corresponding stiffness profile along the red line in (b).

In some cases, there are groups of fibrils that appear to be bundled. These bundles may be distinct from single microfibrils. The red hash marks in Figure 3(a) correspond to the red circles on the line profile and are typically the darkest pixels in the image. The distances between these are measured and tallied as bundles. The green hash marks on the image correspond to the green crosses on the profile and are selected as interfaces between the smallest microfibril that is detected visually on the image or as local minima on the line profile. The distances between these can be measured and tallied as microfibrils. A comparison of topography and transverse stiffness can be seen in Figure 4.

**Figure 5:**
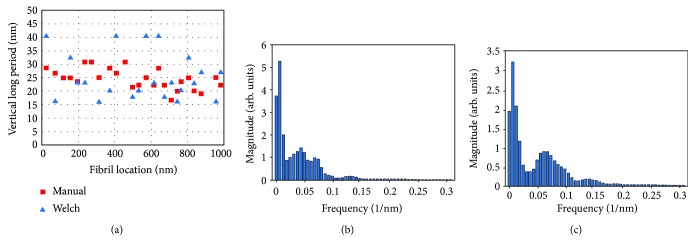
(a) The vertical long period of the SK76 UHMWPE fiber scan in Figure 3(a) established by both visual counting and frequency analysis using the Welch power density spectra method with example spectra seen in (b) and (c) where the tallest peaks indicate the vertical long period frequency.

In addition to the fibril width, features along each fibril can be measured and counted. In order to do this, the full image was inspected to determine which fibrils were continuous through the full length of the image. The fibrils that extend across the entire image are indicated by the black arrows at the top of Figure 3(a) and their position along the profile is indicated by the black dots. In some cases, fibrils may extend the full length of the image but may appear wavy, so a segmented line tool can be used to create a profile along the tops of each fibril. A selected area of Figure 3(a) denoted by the dashed box is magnified in Figure 3(b). This includes the 2 fibrils, numbers 6 and 8, whose line profiles are plotted in Figure 3(c). Only the first 400 nm of the fibrils are shown for clarity, but the analysis was applied to the full line profiles for each of the 25 fibrils selected. Using the Welch discrete power density analysis routine in the MATLAB software package, it is possible to see the frequency peaks of the relevant repeat length of the microstructure, with the results shown in Figures 4(b) and 4(c) (for fibrils 6 and 8, resp.). Welch's technique is a method of determining power spectral density by applying a discrete fast Fourier transform (FFT) to averaged, short periodograms to minimize noise [24]. Visual inspection was used to determine expected nominal repeat unit distances for each fibril. The peak with the highest signal corresponds well with the repeat distance estimated manually through visual inspection of the line profile. An example of the visual inspection is shown in Figure 3(c) as about 25 nm.  Figure 5(a) is a comparison of the manual (red squares) and the peak calculated from the power density spectra method (blue triangle) for each of the fibril long periods measured.

**Figure 6:**
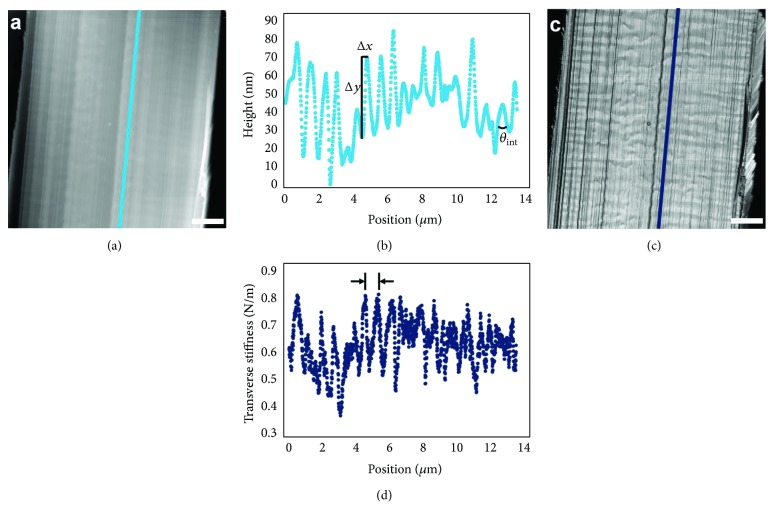
Summary of AM-FM analysis of Kevlar fibers. (a) Representative topography map of K119 fiber. (b) Topographical line scan indicating parameters of interest (Δ*x*, Δ*y*, *θ*_int_). (c) Representative stiffness map of K119 fiber. (d) Transverse-stiffness line scan, highlighting peak-to-peak distance measurable in FFT, equivalent to the thickness of two adjacent stiffness bands. Maps in (a) and (c) were adapted from [17] with permission from Elsevier. Scale bars: 2 *μ*m.

As with UHMWPE, the internal structural motifs of DuPont Kevlar fibers have also garnered tremendous interest within the scientific community over the last several decades. Among many studies investigating Kevlar structural features with various techniques, a seminal study by Dobb et al. provided direct, real-space measurements of microtomed Kevlar K49 fiber cores using dark-field transmission electron microscopy (TEM) [11]. Through in situ TEM tilting, they observed that K49 fiber cores exhibit a “pleated sheet” structure along the fiber axis, with adjacent pleat components approximately 250 nm in length at alternating angles of ±5 degrees from the fiber axis [11]. It would be of great interest to extend this characterization to Kevlar fibers that (i) constitute new Kevlar classes with unique processing conditions and (ii) are not subjected to microtomy, which can yield significant surface artifacts [14]. To address these objectives, AM-FM characterization of FIB-notched samples of four Kevlar fiber classes were performed, exploring connections among fiber processing conditions, internal structures, and tensile properties [17]. Traditional tapping mode topographical maps (Figure 6(a)) of FIB-notched surfaces reveal a pleated structure, which is qualitatively consistent across all Kevlar classes explored (K119 (shown), K29, KM2+, and K49) and correlates well with the TEM studies by Dobb et al. Significant information is also obtained from transverse-stiffness maps (Figure 6(c)) which reveal local material responses to AFM probing throughout the fiber. The trends in these fibers' processing-structure-property relationships are discussed in detail elsewhere [17], while the focus of this work is on the measurement and analysis techniques employed in those studies.

Beyond qualitative observations, Kevlar fibers can be compared most effectively through quantitative measurements of key features observed in topography and stiffness maps. By obtaining point-by-point maps, line profiles (Figures 6(b) and 6(d)) within regions of interest can be directly analyzed. From topographical scans, the lengths of pleat components (L) and the interior angles between adjacent pleat components (*θ*_int_), shown schematically in Figure 5(b), are calculated as:

(1) $$\begin{array}{c} L=\sqrt{{\left(\Delta x\right)}^{2}+{\left(\Delta y\right)}^{2}},\\ \theta_{\mathrm{int}}=180^{{}^{\circ}}-\overset{2}{\underset{i=1}{\sum }}\tan^{-1}\left(\frac{\Delta y_{i}}{\Delta x_{i}}\right), \end{array}$$

where “*i*” is an indexing term for pleat components denoting that adjacent pleat components must be used in the interior angle calculation. From these direct, real-space AFM measurements, [17] it was found that pleat components were normally 200–300 nm long, and pleat interior angles were typically 170–175°, closely matching the observations of Dobb et al. [11].

To quantify spatial variations in stiffness, a one-dimensional FFT code was used to quantify peak-to-peak distances in stiffness map line profiles (Figure 6(d)), with the most commonly detected spatial frequency selected as the average peak-to-peak distance in real space. (This has been selectively verified against real-space measurements using the same technique described earlier and shown schematically in Figures 3 and 5.) From Figures 6(c) and 6(d), it is clear that local transverse-stiffness values can alternate along the pleated microstructure. Each subdomain was classified, with either low or high stiffness, as a stiffness band, and the average stiffness band thickness was calculated as half the peak-to-peak distance computed from FFT. With these techniques combined, AM-FM scans can be used to quantify variations in inherent fiber topography and transverse stiffness, advancing understanding of internal Kevlar fiber morphologies.

#### 3.1.2. Image Processing Tools for Quantifying Microstructure Populations

**Figure 7:**
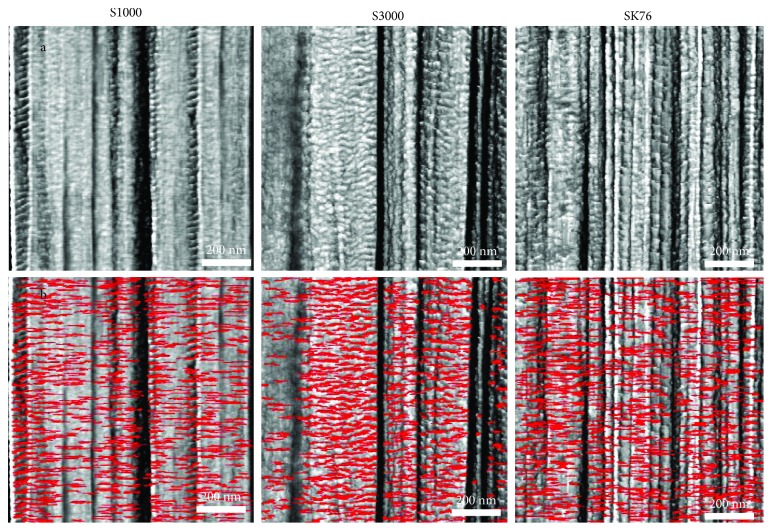
(a) UHMWPE greyscale AFM stiffness map of internal surfaces of S1000, S3000, and SK76 commercial fibers, where the darker shading is the more complaint, disordered material and the lighter shading is the stiffer, more crystalline material. (b) Duplicate stiffness maps of the ones above are shown, with lamellae highlighted in red by the spatial derivative of stiffness.

To demonstrate the capability of characterizing high-performance materials, the microstructures of various ballistic fibers were quantified using simple scripts and image processing routines in the MATLAB software package. Distinct morphologies can be seen in the transverse-stiffness AFM scans of three commercial UHMWPE fibers, S1000, S3000, and SK76 (Figure 7(a)). To reiterate, the transverse-stiffness scan is sensitive to the local composition of the material, and in the case of UHMWPE provides excellent contrast between stiffer, more crystalline regions indicated by the lighter shading, and more compliant, disordered regions indicated by darker shading. It is easy to visually identify a repeating dark/light lamellar structure along the length of the fiber that resembles the stripe patterns on a tiger. This repeating microstructure is critical to understanding the mechanical response of a given fiber and can be quantified with visual processing techniques. To assist in highlighting the edges of a lamellar structure and provide a boundary for visual processing algorithms to analyze, the discretized spatial derivative of transverse stiffness is taken pixel-by-pixel across the AFM scan images using a fourth-order central difference formulation:

(2) $$\begin{array}{c} \frac{\partial k_{T}}{\partial x_{i}}≅\frac{1}{12L_{pixel}}\left[-k_{T}\left(x_{i}+2L_{pixel}\right)+8k_{T}\left(x_{i}+L_{pixel}\right)+k_{T}\left(x_{i}-2L_{pixel}\right)-8k_{T}\left(x_{i}-L_{pixel}\right)\right]+O\left({L_{pixel}}^{4}\right), \end{array}$$

where k_T_ is the transverse stiffness, *x* is the spatial pixel location where the derivative is being evaluated, the subscript *i* denotes which spatial direction the partial derivative is taken with respect to (*x* or *y*), and L_pixel_ is the length of a pixel. The resulting derivative field is thresholded to remove noise and overlaid in red on the AFM scans (Figure 7(b)), with only the positive-valued derivatives displayed for simplicity of viewing.

**Figure 8:**
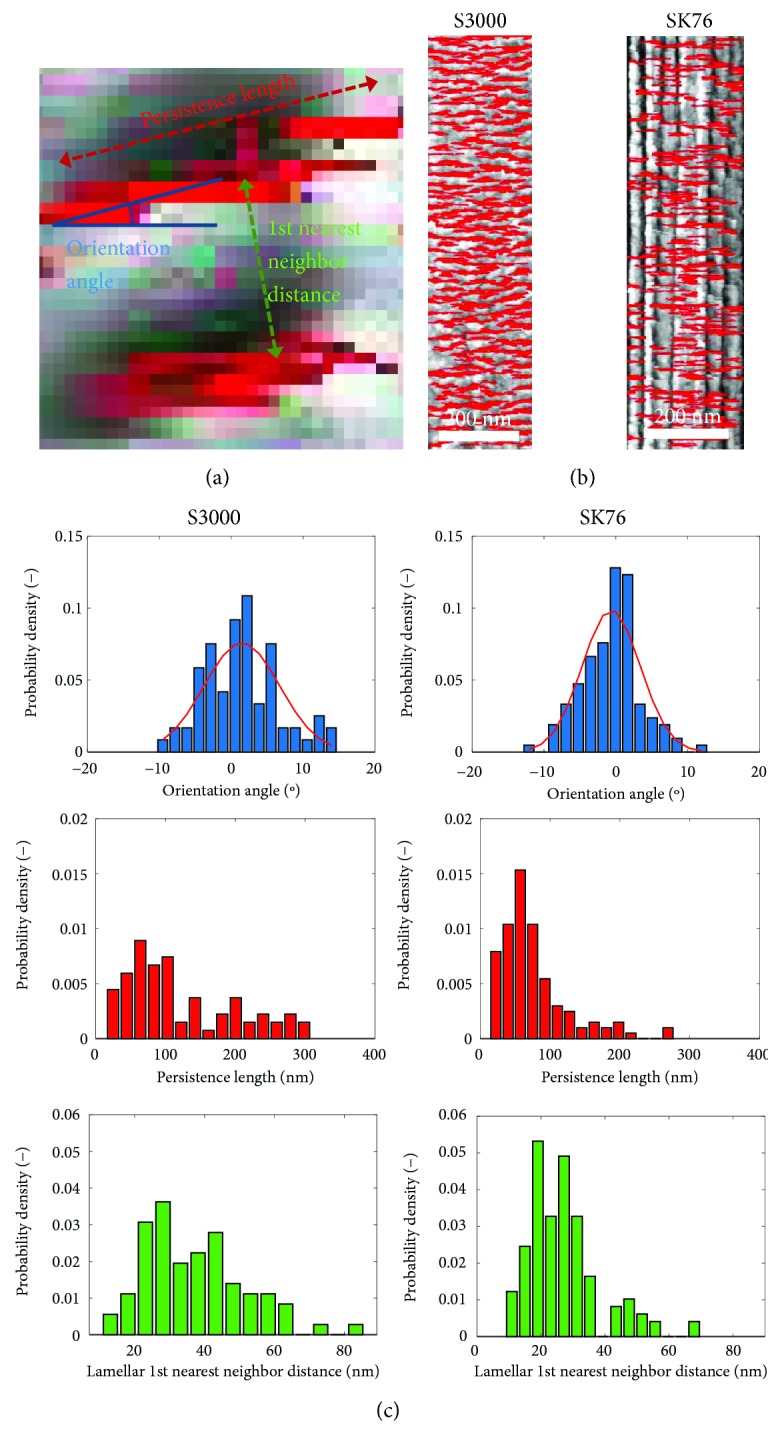
(a) Schematic showing the lamellar persistence length, orientation angle, and first nearest neighbor metrics for UHMWPE microstructural analysis, (b) magnified sections of the S3000 and SK76 in Figure 7(a), and (c) the corresponding histograms of the three metrics for both sections of magnified fibers.

The overlaid spatial derivative of stiffness dramatically highlights the contrasting morphological differences between the fibers. In the S1000 scan, it is evident that there are regions of fibrils where the lamellae are almost perfectly perpendicular to the fiber axis direction. S3000 and SK76, on the other hand, show lamellae that are angled much more irregularly. The three metrics of interest for quantifying microstructure in UHMWPE are (i) orientation angle (the axis perpendicular to the fiber axis is defined as 0°), (ii) the persistence length, which is the overall length of the highlighted lamellar domains, and (iii) the first nearest neighbor distance, which is defined as the distance between a reference lamella and its closest nearest neighbor, as depicted in Figure 8(a). These three metrics are immensely useful for quantifying the microstructure of UHMWPE and comparing fibers that have been processed in different conditions to yield different mechanical properties. The red highlighted lamellar domains in Figure 7(b) are analyzed by fitting an ellipse to each individual domain using built-in image processing routines in MATLAB. The ellipses' orientation angle, persistence length (measured by major axis length), and first nearest neighbor distance (measured by taking a reference ellipse and measuring the centroid-to-centroid distance to the closest neighbor) are then trivial to measure across all detected lamellae in the processed AFM scan and can be summed.

To illustrate the measurable differences in fiber morphology, representative sections of the fiber scans (depicted in Figure 8(b)) are analyzed and compared through histograms shown in Figure 8(c). The region depicted in Figure 8(b) of the S3000 fiber was selected because of the lack of a clear fibrillated structure, whereas SK76 was selected because of the clear vertical fibrils. Within those magnified regions, there are clear differences between S3000 and SK76 as shown by the histograms of the three morphological metrics of interest. The SK76 fiber has a narrower distribution of orientation angle, a lower average lamellar persistence length, and a slightly lower nearest neighbor distance than the S3000 fiber.

**Figure 9:**
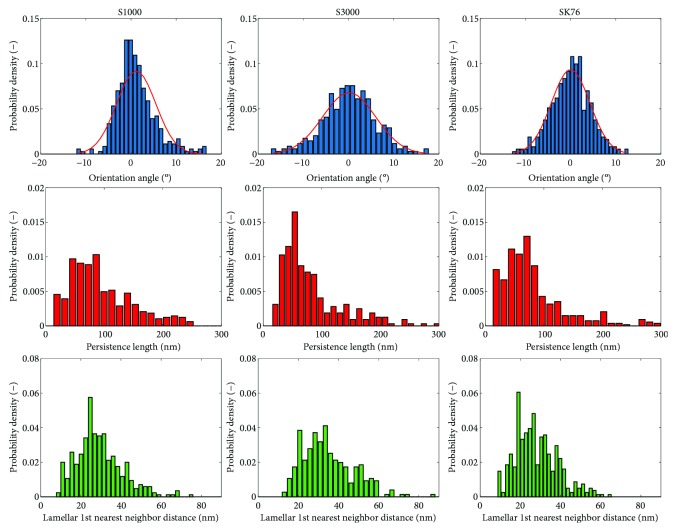
Histograms of orientation angle, persistence length, and 1st nearest neighbor distance between lamellae determined by visual processing of the AFM scans in Figure 6 for S1000, S3000, and SK76 UHMWPE fibers.

The results of the same analysis across the entire scans of the fibers in Figure 6 can be seen in the histograms of Figure 9, with the mean values shown in Table 1. S3000 has a broader orientation angle histogram than SK76 (calculated by the standard deviation of the distribution), indicating that there is less alignment of the crystal lamellae in S3000. The persistence length of the lamellae is also longer in S3000 than SK76, suggesting that the crystalline order carries over longer lengths in S3000. Finally, the nearest neighbor values for SK76 are smaller than S3000, suggesting thinner lamellae, however all values of the nearest lamellae correspond well with the repeat distances calculated by the earlier Welch power density frequency analysis (Figure 5(a)), about 20–35 nm in vertical lamellar repeat distance. The results of the analysis of the smaller, representative region and the analysis of the entire scans are consistent, as summarized in Table 1.

## 4. Conclusion

**Figure 10:**
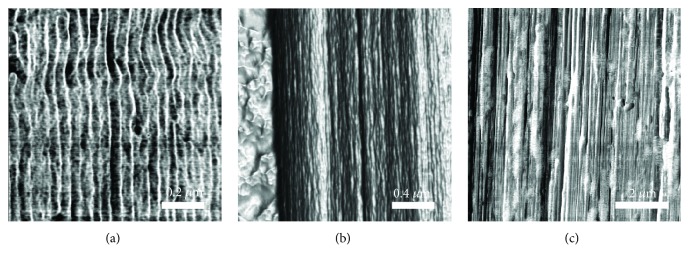
Representative AFM maps of selected high-performance materials. (a) Cotton fiber stiffness map. (b) *Bombyx mori* silk fiber stiffness map. (c) SSE-UHMWPE phase map. Materials were provided through the courtesy of (a) Natick Soldier Research, Development, and Engineering Center (NSRDEC), (b) Bolt Threads Inc., and (c) DuPont.

The analysis techniques described in this work can identify clear differences in microstructure in materials, as shown by example analysis of high-performance polyethylene and para-aramid commercial fibers. To demonstrate sensitivity to processing history, three different types of commercial UHMWPE fibers were compared, ensuring that each sample was produced from different processing conditions. Using an AFM scanning technique to interrogate micron-size areas of a given sample, local phase variations in the material could be detected with nanometer resolution, showing a rich mosaic of crystalline and amorphous domains. Analysis of these AFM scans revealed significant differences in the distribution and arrangement of these morphologies, giving a so-called fingerprint of the fibers and their processing conditions. As a technique benchmark, the same AFM scanning method revealed the well-established pleat structure when applied to commercial Kevlar fibers. While the visual processing methods described in this work are demonstrated with AFM scans, they can easily be applied to spatially resolved images like SEM or TEM, albeit with the resolution and sample preparation limitations described in the Introduction. Furthermore, these techniques can be applied to a variety of materials of interest to elucidate the impact of microstructure on mechanical performance, ranging from natural materials like cotton and spider silk, to solid state extruded (SSE) UHMWPE (Figure 10).

Achieving superior properties in a material requires an understanding of how process conditions impact microstructure, as well as the relationship between microstructure and performance. Critical to bridging these requirements is a reliable method of quantifying morphology within materials at small scales. With high-resolution, material-phase-sensitive spatial scanning techniques like AFM, coupled with knowledge of the molecular organization of these materials, it is possible to measure constituent morphologies and local phase distributions that are critical for establishing models which can predict mechanical properties.

## Figures and Tables

**Table 1:** Mean values for the relevant morphological metrics of S1000, S3000, and SK76 UHMWPE fibers.

Fiber	Mean values from full domain (Figure 7)			Mean values from small domain (Figure 8)		
	Orientation angle (°)	Persistence length (nm)	1st NN distance (nm)	Orientation angle (°)	Persistence length (nm)	1st NN distance (nm)
S1000	1.15 ± 4.34	95.6	29.2	—	—	—
S3000	0.12 ± 5.86	87.9	34.6	1.48 ± 5.25	121.1	36.9
SK76	0.05 ± 3.41	82.3	23.0	−0.65 ± 4.03	75.1	27.2

## Data Availability

The images used to support the findings of this study are included within the article itself and the supplementary information file(s).

## Supplementary Materials

Supplementary Materials Three separate codes utilized in the evaluation of the stiffness data can be found in the Supplementary Materials.
