# Supplementary material for: Quantifying High-Performance Material Microstructure Using Nanomechanical Tools with Visual and Frequency Analysis
Source: Scanning. 2018 Jul 12;2018:4975317. doi: 10.1155/2018/4975317 (PMC6079397; doi:10.1155/2018/4975317)
Supplement: Supplementary Materials — Three separate codes utilized in the evaluation of the stiffness data can be found in the Supplementary Materials. [file 4975317.f1.pdf]

Supplemental Information

Quantifying high-performance material microstructure using nanomechanical tools with visual and frequency analysis

Emil Sandoz-Rosado[1], Michael R. Roenbeck[1], Kenneth E. Strawhecker[1]*

[1]Composites and Hybrid Materials Branch, US Army Research Laboratory
*4600 Deer Creek Loop, RDRL-WMM-A, Aberdeen Proving Ground, MD 21005, 410-306-1342, kenneth.e.strawhecker.civ@mail.mil

Ruling out scan angle and topography for the cause in stiff/complaint regions along the length of the fibril

We performed and analyzed a scan on a commercial ultra-high-molecular-weight-polyethylene (UHMWPE) fiber at roughly 45° scan angle and saw the same morphologies with an appropriate angular tilt (Supplemental Figure 1). The highlighted domains in Supplemental Figure 1 show bands that are perpendicular to the fiber direction, despite being at a 45° scan angle. This suggests that the AFM scan angle does not create an artifact that generates the stiff and compliant regions that are perpendicular to the fiber direction.

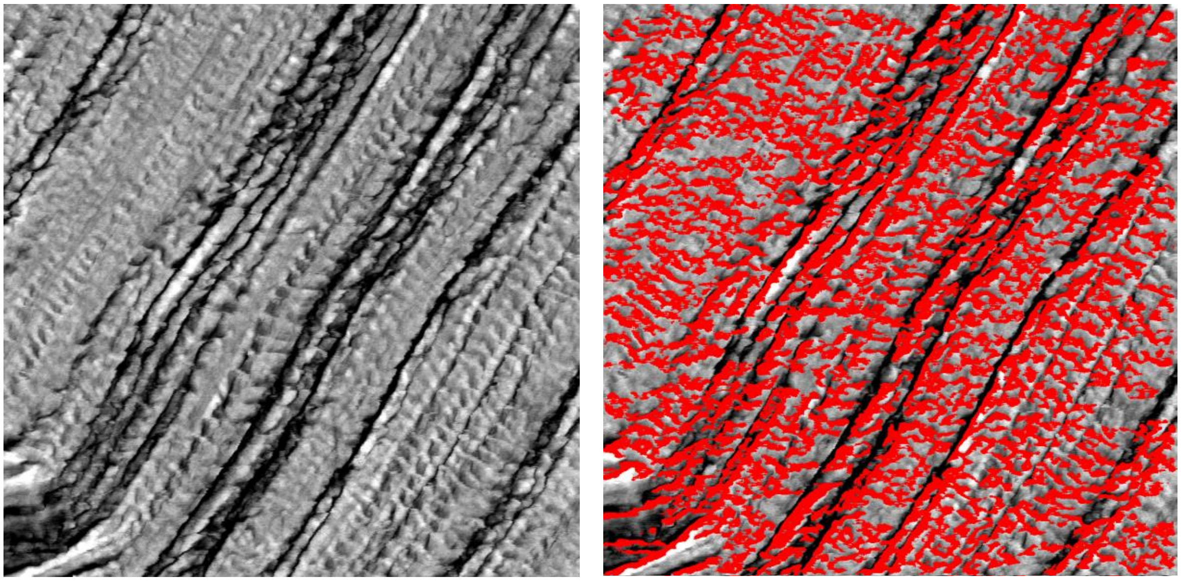

Supplemental Figure 1: AFM AM/FM stiffness scans UHMWPE fiber, scan size is 1μm in length and width (left) unprocessed scan (right) scan analyzed with central difference algorithm showing banding perpendicular to the fiber direction.

Sensitivity to curvature between the tip and local asperities is unlikely to be the dominant cause the cause of the differences in stiffness measured by the AM/FM technique along a fibril in UHMWPE, as the following Hertzian theory demonstrates. Hertz theory provides analytical solutions for the measured stiffness for the indentation extremes: a) a hemisphere indenting a hemisphere of comparable diameter

(large relative slope difference) and b) a hemisphere indenting a flat surface (small relative slope difference), as seen in Response Figure 1.

The expression for measured stiffness of the hemisphere-on-hemisphere (subscript *h-h*) is [1]:

$$k_{h-h} = \frac{P}{\alpha} = \frac{2}{(3\pi)^{\frac{2}{3}}}(V_1 + V_2)^{-\frac{2}{3}}\left(\frac{1}{D_1} + \frac{1}{D_2}\right)^{-\frac{1}{3}} P^{\frac{1}{3}}$$

where k is the contact stiffness, P is the contact force, α is the total elastic compression at the point between two bodies, V is the elastic parameter equal to $(1-\upsilon^2)/\pi E$ (where υ is Poisson's ratio and E is elastic modulus), D is the diameter of the hemisphere, the subscripts 1 and 2 represent the two different domains in contact. Conversely, the expression for contact stiffness between a hemisphere indenting a flat semi-infinite plane (subscript *h-f*) is [1]:

$$k_{h-f} = \frac{P}{\alpha} = \frac{2}{(3\pi)^{\frac{2}{3}}}(V_1 + V_2)^{-\frac{2}{3}}(D_1)^{\frac{1}{3}} P^{\frac{1}{3}}$$

with this special case having D_1 be the diameter of the single hemisphere indenting the flat plane. Taking the reviewer's example of the two hemispheres having equal diameter ($D_1 = D_2 \sim$ 40nm), the ratio of the stiffness $k_{h-f} / k_{h-h} = 2^{1/3} = 1.26$, so the difference in stiffnesses between regions with vastly different curvatures only amounts to about 26%, while the measured stiffness of a stiffer region is 2-4 times stiffer than the measured stiffness of the more compliant regions along the length of the same fibril. This suggests that there is another explanation for the discrepancy in stiffness, and it is likely a difference in material composition, density or phase. Finally, in Figure 7a, the S3000 has a region on the left portion of the scan that does not have much contrast in stiffness along the length of the fiber, despite having similar topography to the rest of the fiber- this is an example of a more homogeneous section of the fiber.

Raw images for analysis

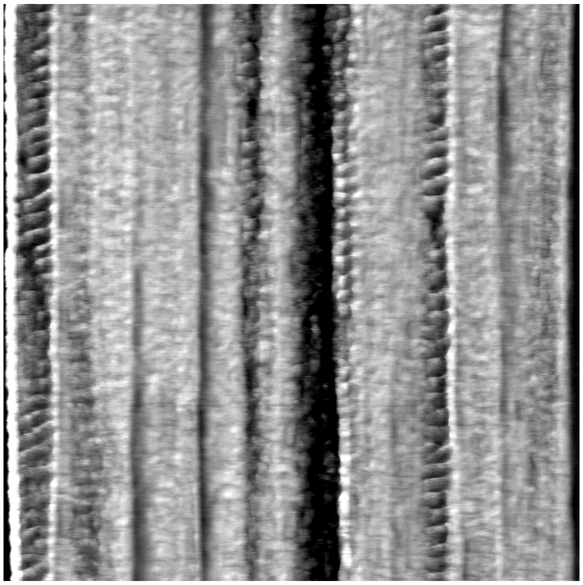

Supplemental Figure 2: S1000, 1um stiffness AFM scan (used in Figure 7 and Figure 8)

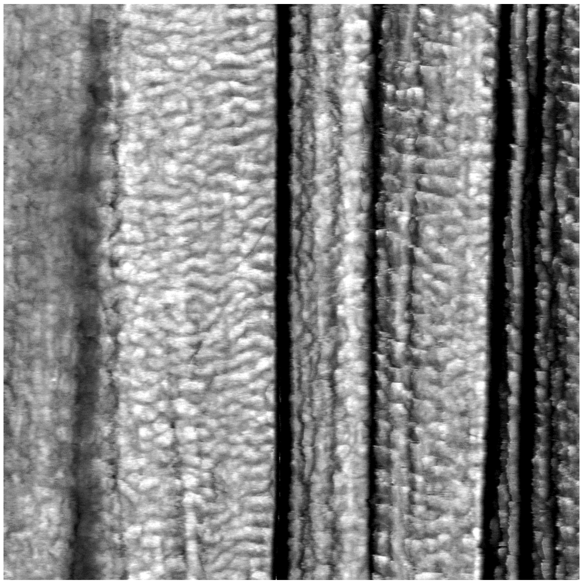

Supplemental Figure 3: S3000, 1um stiffness AFM scan (used in Figure 7 and Figure 8)

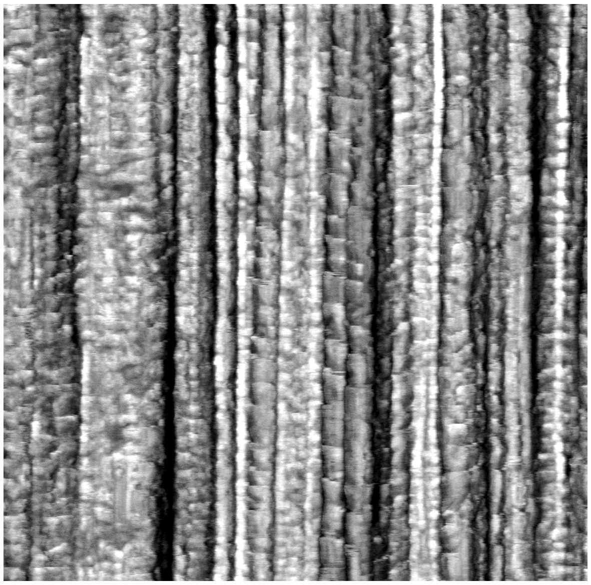

Supplemental Figure 4: SK76, 1um stiffness AFM scan (used in Figures 3,4,7 and 8)

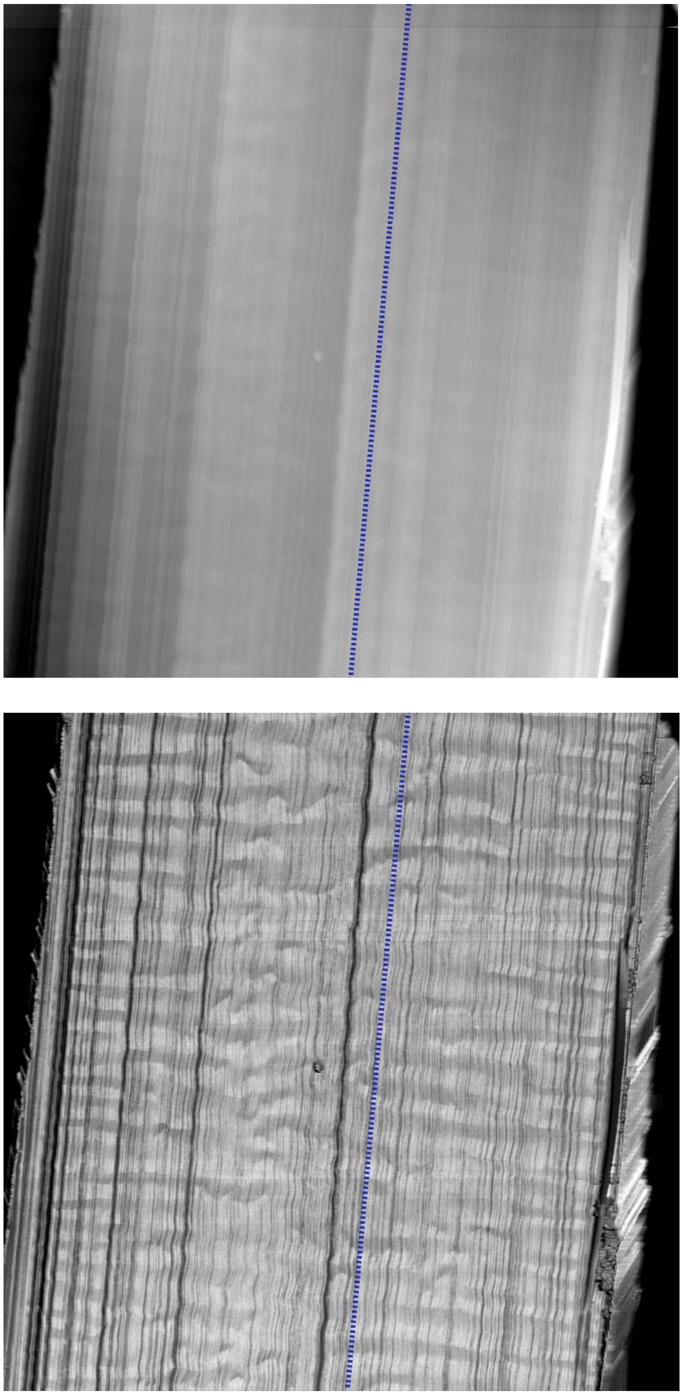

Supplemental Figure 5: Kevlar® K119, 14um (top) topography and (bottom) stiffness AFM scan (used in Figure 6)

References

[1] Puttock, M.J. and Thwaite, E.G., “Elastic compression of spheres and cylinders at point and line contact” National Standards Laboratory Technical Paper No. 25, Commonwealth Scientific and Industrial Research Organization, Australia, 1969
